# Object-based attention during scene perception elicits boundary contraction in memory

**DOI:** 10.3758/s13421-024-01540-9

**Published:** 2024-03-26

**Authors:** Elizabeth H. Hall, Joy J. Geng

**Affiliations:** 1 Department of Psychology, University of California Davis, Davis, CA 95616 USA; 2 Center for Mind and Brain, University of California Davis, Davis, CA 95618 USA

**Keywords:** Boundary extension, Selective attention, Scene perception, Object affordances

## Abstract

Boundary contraction and extension are two types of scene transformations that occur in memory. In extension, viewers extrapolate information beyond the edges of the image, whereas in contraction, viewers forget information near the edges. Recent work suggests that image composition influences the direction and magnitude of boundary transformation. We hypothesize that selective attention at encoding is an important driver of boundary transformation effects, selective attention to specific objects at encoding leading to boundary contraction. In this study, one group of participants (*N* = 36) memorized 15 scenes while searching for targets, while a separate group (*N* = 36) just memorized the scenes. Both groups then drew the scenes from memory with as much object and spatial detail as they could remember. We asked online workers to provide ratings of boundary transformations in the drawings, as well as how many objects they contained and the precision of remembered object size and location. We found that search condition drawings showed significantly greater boundary contraction than drawings of the same scenes in the memorize condition. Search drawings were significantly more likely to contain target objects, and the likelihood to recall other objects in the scene decreased as a function of their distance from the target. These findings suggest that selective attention to a specific object due to a search task at encoding will lead to significant boundary contraction.

## Introduction

More than 30 years ago, Intraub and Richardson (1989) reported a consistent pattern of errors in memory for scene photographs where people remembered more details than were actually present in the original picture. Since then, this pattern of errors, dubbed "boundary extension," has been replicated in numerous studies (Candel et al., 2004; Chadwick et al., 2013; Chapman et al., 2005; Green et al., 2019; Intraub et al., 2008; Kong et al., 2010; Lin et al., 2022; Mathews & Mackintosh, 2004; McDunn et al., 2014; Munger & Multhaup, 2016; Park et al., 2007; Patel et al., 2022; Seamon et al., 2002; Wan & Simons, 2004). In a subsequent study, Intraub et al. (1992) found a similar, yet inverse pattern of errors in memory—participants would sometimes remember the boundaries of wide-angle pictures as being more constricted than they originally were. Similar studies showed that contraction and extension are not fixed features of scene images but can be adjusted based on the viewing distance of single images or manipulating the image set (Chadwick et al., 2013; Intraub et al., 1992; McDunn et al., 2016; Ménétrier et al., 2018). Building on these studies, we test whether the task-induced distribution of attention during scene viewing contributes to the type of boundary transformation that occurs for an image in memory.

A recent resurgence of research on boundary transformations has begun to examine how different image properties influence boundary transformations in memory. Bainbridge and Baker (2020) tested more than 1,000 images in 2,000 participants and found that pictures of close-up, central objects tend to elicit extension, while far-away image viewpoints of scenes with distributed objects tend to elicit contraction. This suggests that the distance of the image viewpoint focusing on a single object or an entire scene determines whether scene contraction or extension occurs in memory (Hafri et al., 2022). Going further, Park et al. (2021) tested participants on a set of stimuli that varied in both the viewpoint distance and the number of objects in each environment. They reported that the distance *transition point* at which participants reliably experienced neither contraction nor extension in memory varied by the objects present in the scene. Scenes populated with many small and manipulable objects elicited closer transition points in memory, indicating a bias towards contraction, but scenes that contained only a few, large, and space-defining objects, like tables and bookshelves, elicited farther *transition points* in memory. These findings suggest that if participants were focusing on an object present in the scene, their memory would be biased to the best viewpoint to process the object. Whereas if the participants were focusing on the scene as a whole, their memory would be biased to the best viewpoint to process the scene identity.

Consistent with the notion that object processing impacts the degree of boundary extension or contraction, emotional and semantic object properties can also drive boundary transformations. For example, scenes with negatively valent objects, like weapons or graphic injuries, will limit the degree of extension and can even elicit significant contraction effects in memory (Christianson, 1984; Christianson & Loftus, 1987; Green et al., 2019; Ménétrier et al., 2013; Safer et al., 1998, 2002; Takarangi et al., 2016; Wonning, 1994). This presumably occurs because high valence objects capture attention and focus image processing on a single object. Similarly, images with heterogenous object semantics elicit more contraction in memory, compared with scenes that contain the same amount of shared semantic label objects (M. Greene & Trivedi, 2022). This effect is most likely due to related objects being automatically attended together, leading to more distributed attention over the scene image (Mack & Eckstein, 2011; Nah & Geng, 2022; Nah et al., 2021; Wei et al., 2018). These results suggest that the objects participants attend to during perception is an important factor in determining the trend and degree of transformation.

Together, the literature suggests that image properties and object content both impact scene memory. One possible explanation for these differences is that the images may lead to systematic differences in how attention is distributed during scene encoding (Intraub et al., 2008). Following along the line of work done by Park et al. (2021), we hypothesized that instructing participants to find and encode a small object in a wide-angle scene may lead to a shift in memory towards boundary contraction because the target object is misremembered at a more preferential viewpoint for processing its identity (i.e., closer than it originally appeared). Likewise, instructing participants to process and encode the identity of the “scene” within the wide-angle image would lead to participants misremembering the image to a viewpoint that was preferential for processing scene identity.

In this current study, we show two groups of participants the same scene images but ask them to either engage in target search, or simply memorize the image. We hypothesize that those engaged in target search will focus attention primarily on the target object (Wu & Wolfe, 2022; Young & Hulleman, 2013; Yu et al., 2022), leading to a higher degree of boundary contraction in memory; in contrast, those engaged in scene memorization only will distribute attention more broadly, leading to less boundary contraction. The influence of image composition is kept constant across groups by using the same images, but the goals of the viewer are manipulated by task. After encoding, all participants are given a surprise drawing task in which they are asked to draw as many of the previously seen scenes as possible. Their drawings were analyzed for the objects recalled, their drawn location and size, and the transformation of scene boundaries. Results revealed significant boundary contraction in drawings from participants who engaged in search, while drawings from the memorize condition showed equal rates of contraction and extension. Further analyses of boundary contracted search drawings revealed diminishing memory for objects as a function of distance from the target object, whereas the smaller amount of memorize drawings that exhibited contraction revealed diminishing memory for objects as a function of the distance from the center of the image. These findings provide evidence that boundary transformations in memory are due to how attention is distributed amongst objects at encoding.

## Method

### Participants

Thirty-six undergraduate students (26 females, mean age = 19.44 years, *SD* = 1.34, range: 18–23 years) participated in the search condition, and 36 different students participated in the memorize condition (27 females, mean age = 19.94 years, *SD* = 1.67, range: 18–25 years). Students were recruited from the University of California, Davis, through the Sona research pool in exchange for research credit. Participants were native English speakers with normal or corrected vision. We also recruited online scorers to judge the drawings on a variety of metrics. Five-hundred and seventy-nine scorers were collected from Amazon Mechanical Turk and were monetarily compensated. One hundred and sixty-four scorers were collected from the SONA research pool to complete ratings on Testable for course credit. Each participant provided informed written consent in accordance with the local ethics clearance as approved by the National Institutes of Health.

### Stimuli

**Fig. 1:**
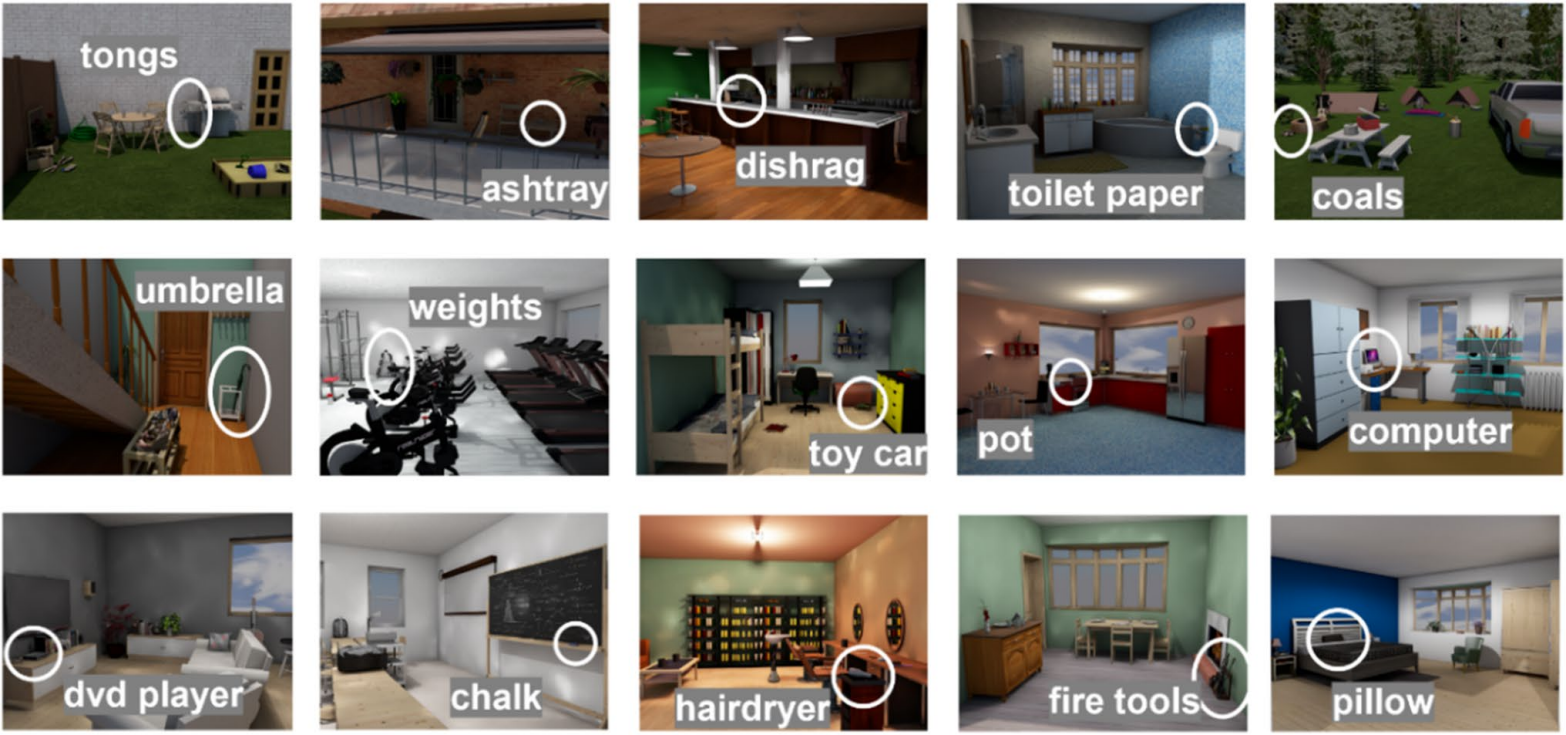
The 15 scene images studied by participants in the search and memorize conditions. In the search condition, participants were instructed to find the circled target object

The 15 scene images used in this study were initially constructed for an experiment assessing the role of anchor objects on eye movements in visual search (Boettcher et al., 2018). The stimulus images were created with ArchiCAD software version 18 (Graphisoft, Munich, Germany). All images were 1,280 wide by 960 pixels tall. Each scene contained a visual search target (e.g., toilet paper). The scenes were selected so that there was no overlap in target objects across the scenes (Fig. 1), and each scene could be identified by a unique categorical identifier (i.e., there was only one kitchen in our stimulus set).

### Apparatus

Stimuli were presented on a ASUS MG279Q monitor with a 60-Hz refresh rate and a spatial resolution of 1,920 × 1,200 pixels. Participants were seated 60 cm away from the screen and a computer running PsychoPy (Peirce, 2007) controlled all stimulus presentations. Eye movements were tracked using an EyeLink-1000 desktop mount, sampling from the right eye at 500 Hz (SR Research, Ontario, Canada).

### Experimental design

**Fig. 2:**
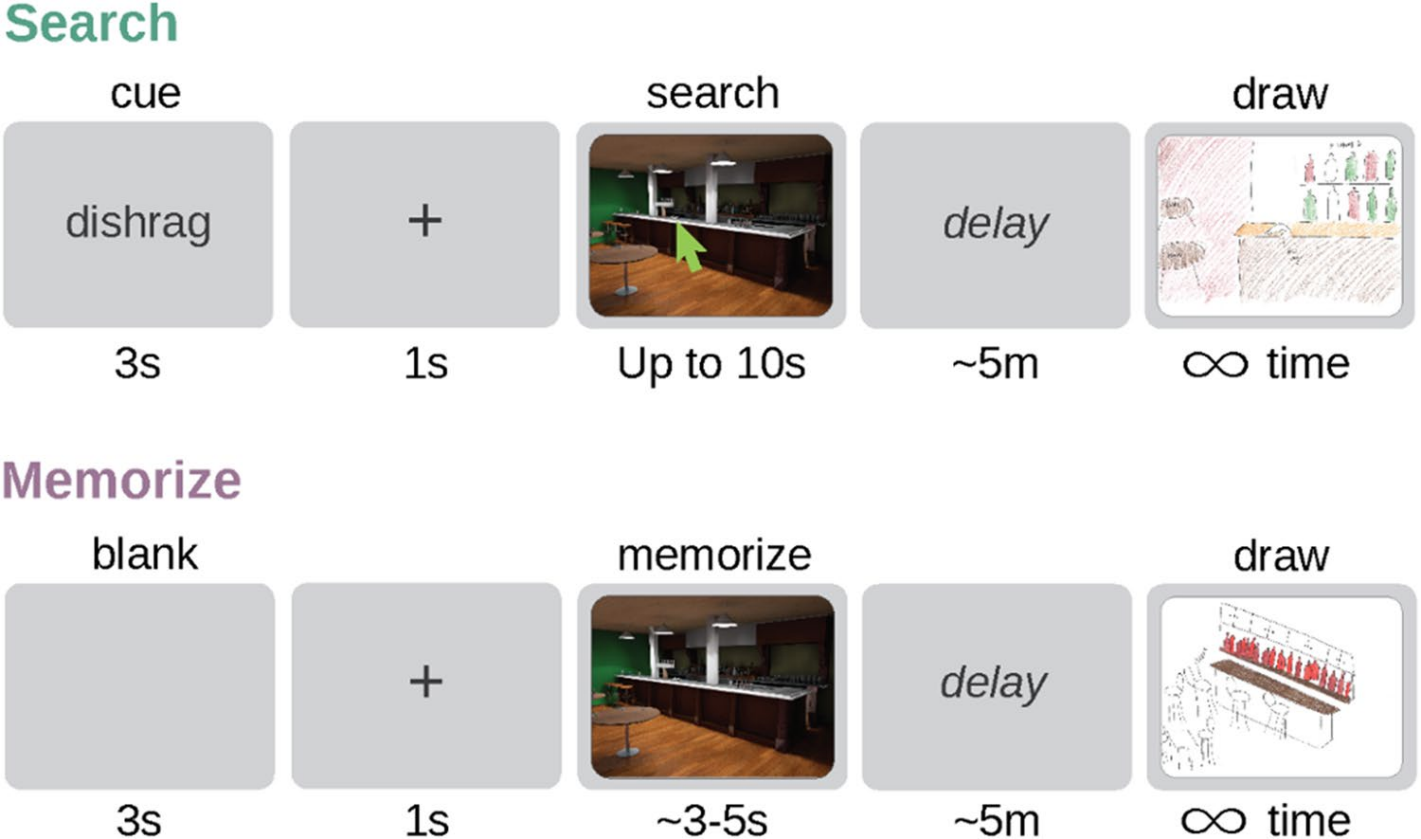
In the search condition, participants (*N* = 36) were given target cues and had up to 10 seconds to find the cued objects in 15 scene images. In the memorize condition (*N* = 36), participants memorized each scene for the average amount of time it was viewed by participants in the search condition. After a delay, both groups of participants had an unlimited amount of time to draw the scenes from memory. (Color figure online)

The visual search group was run before the memorize group so that scene exposure times from the search group could be used to constrain viewing time in the memorize group. All saw the same 15 computer-generated scenes, and both groups completed one practice trial with a scene that was not from the main experimental set (Fig. 2). The search group was instructed to search for and click on a specific target. Each trial, first the target cue word appeared on the screen for 3 s, followed by a 1-s fixation cross, after which the stimulus image appeared on the screen. Once the image appeared, they were given up to 10 seconds to click on the target with the computer mouse. They were also instructed to memorize the scene in as much detail as possible since their memory for the images would later be tested, though specific details of the memory test were not provided.

Participants were then asked to complete the Visual Vivid Imagery Questionnaire (VVIQ), which contains questions regarding their ability to visualize images (Marks, 1973). This task was used to limit rehearsal of the scenes and items in memory and an average of 4.56 minutes (*SD* = 1.48 minutes) passed from the end of the eye-tracking phase to the start of the drawing phase.

After the VVIQ, participants were instructed to draw as many scenes as they could recall, in no particular order and with no time limit, while their pen movements were tracked on a digital drawing pad. They were provided with 15 sheets of paper each with a 1,280 × 960 black frame and were instructed to draw every detail they could remember about the scene within the frame. They were told that the drawings would not be scored on the basis of their drawing ability but would be scored on how accurately they were as a representation of the studied stimulus images. If they felt they could not accurately draw an item in the scene, they were instructed to try to draw the general shape, and they could label anything they felt was unclear. They could use color pencils to add any color they remembered.

In the memorize group, participants were instructed to memorize each scene in as much detail as possible as their memory would be tested later on, and they saw each scene for the average time that the participants viewed the scene in the search experiment (*M* = 4.15s, *SD* = 0.96 s, MIN = 3.23 s, MAX = 6.06 s). They were not instructed to search for the target or click the image and did not see the target word before each image but instead saw a blank screen for 3 s followed by a 1-s fixation cross. An average of 4.22 minutes (*SD* = 1.33 minutes) passed from the end of the eye-tracking phase to the start of the drawing phase.

### Eye-tracking analysis

Fixations and saccades were defined from raw eye-tracking data using the Saccades package in R (von der Malsburg, 2015). Fixations could not be determined for one participant from each condition due to poor data quality. We included the drawings from these two participants in analyses but discarded their eye movement data. We computed the percentage of the scene that was foveated by a participant by placing a circular filter with a 1 degree of visual angle radius centered on each fixation. We defined the percentage of the scene that was foveated in a trial as the summed area of pixels occupied by the circular filters divided by the total amount of pixels in the image (Castelhano et al., 2009).

## Online scoring procedures

The 72 in-lab participants drew 601 scenes from memory. Three scorers, the first author and two undergraduate research assistants, matched each drawing to a scene image. A drawing was considered to be matched to an image if two out of three scorers agreed. If the scorers believed that a participant drew the same stimuli image more than once, the first drawing of that scene was considered a match, and subsequent drawings of the same image were not included in analyses. Drawings that were not matched to an original image by the experimenters were not scored (86 out of 601 drawings, or 13.64% of drawings), leaving 515 drawings for analyses. Of the 86 unmatched drawings, 32 were of the practice trial image. Three different measures were collected for each drawing. The code for these measures was adapted from Bainbridge et al. (2019).

### Boundary transformation

Forty-four scorers were recruited from the SONA research pool to provide ratings of boundary transformation for each drawing on Testable. Scorers were shown the drawing and the originally viewed stimulus image side by side on the screen. Scorers were asked whether the drawing was “closer, the same, or farther than the original photograph,” and were told to ignore any extra or missing objects in the drawing. Scorers responded on a 5-item scale: *much closer*, *slightly closer*, *the same distance*, *slightly farther,* and *much farther*, with the additional option to indicate “can’t tell” if they believed the drawing to be incomprehensible. Seven scorers provided boundary ratings for each drawing and boundary transformation scores for each drawing were calculated by the mean across the ratings normed on a scale of −1 (*much farther*) to +1 (*much closer*).

### Object marking

One hundred and twenty scorers were recruited from the UC Davis SONA research pool to complete an online object marking task on Testable. The purpose of this task was to determine if an object from the original image was included in the drawing or not. Scorers were shown the original image with an object outlined in red using the LabelMe annotations presented next to a drawing. Scorers were asked to indicate if the outlined object was included on the drawing. Scores were collected from three participants per object and an object was determined to be in the drawing if at least two out of three participants agreed that it was present.

All objects in the stimulus images were segmented using LabelMe, an online object annotation tool (Russell et al., 2008). There were 360 objects in the stimulus set and each image contained 24 objects on average (SD = 16.99, min = 8, max = 83). Objects were “nameable, separable, and visually distinct items” (Bainbridge et al., 2021, p. ). If multiple objects of the same type were touching, these objects were grouped together and given a plural label (e.g., “shampoos”). Object parts (e.g., “tire” on truck) were not segmented, but if an object was visually distinct and could be defined as a separate semantic label it was segmented separately (i.e.. decorative “pillow” on a couch). Background segmentations (“grass,” “trees,” “floor,” “walls,” “ceiling”) were not included in analyses (Bainbridge et al., 2019).

### Object location and size

Five-hundred and seventy-nine scorers were recruited on Amazon Mechanical Turk to complete an online object location task. The purpose of this task was to quantify the location and size of drawn objects. Only objects that had been determined to be present in the drawing by the object marking task were scored in this task. Scorers were shown an original image with an object outlined in red next to a drawing and asked to place and resize an ellipse around the same object in the drawing. Three scorers were asked to locate each object of interest in a drawing. Object location was calculated as the median centroid of the ellipses across the responses. Object size was calculated as the median radii of the ellipses across responses.

## Results

### Object-based attention elicits more boundary contraction

**Fig. 3:**
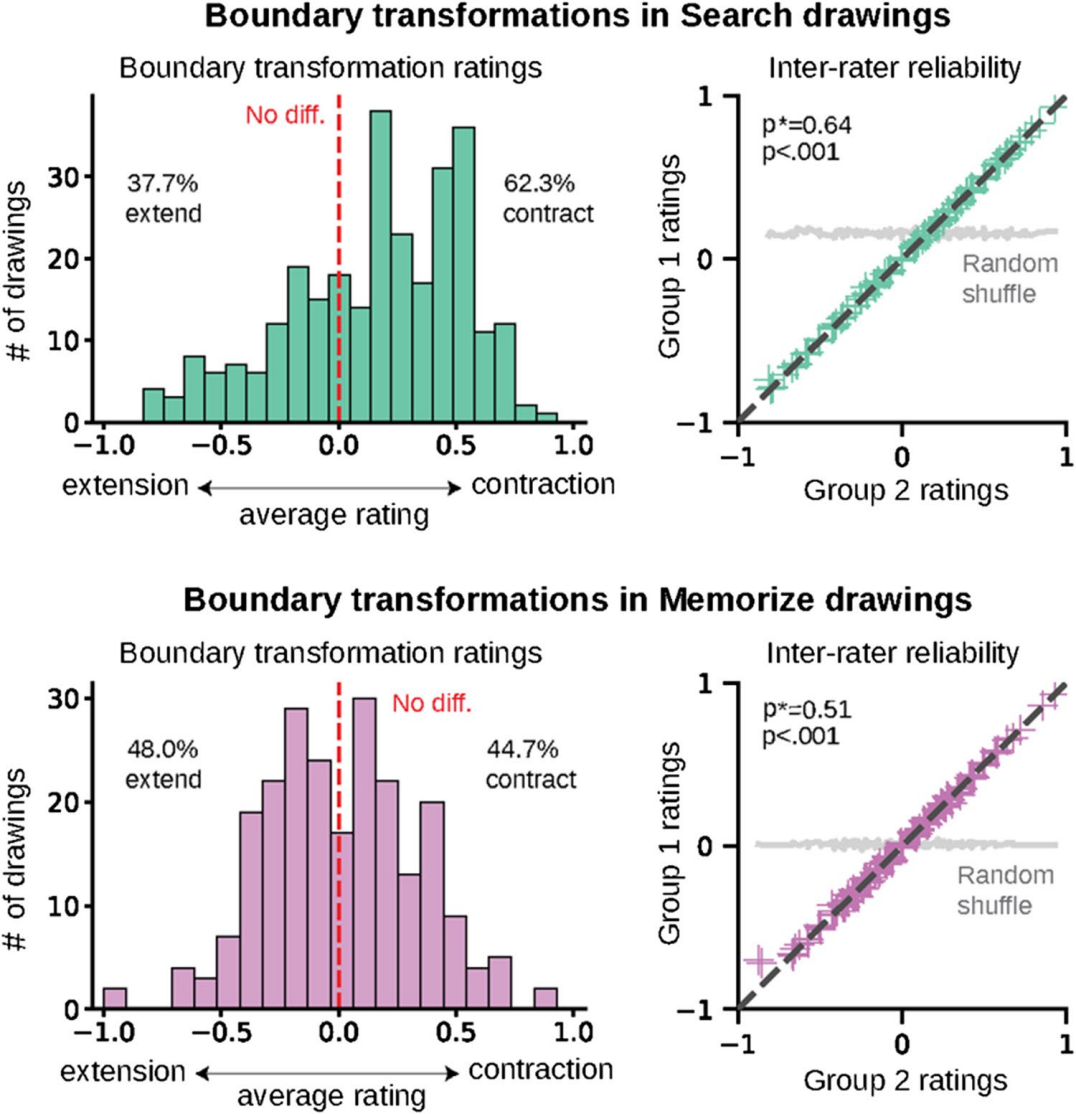
(Left) Histograms of boundary transformations in the memory drawings by condition. On average, 62.3% of drawings in the search condition showed boundary contraction, a significantly greater proportion than the 37.7% that showed extension. Only 44.7% of drawings in the memorize condition showed contraction, while 48.0% showed extension, and there was no significant difference between the proportions. (Right) Results of the split-half consistency analyses for each condition. Seven different raters scored the amount of boundary transformation in each drawing. Each set of ratings was split in half, and we calculated the correlation between the average transformation score of each half. The gray line shows the other half of ratings sorted randomly. For both conditions, ratings between groups were highly similar and significantly correlated. (Color figure online)

**Fig. 4:**
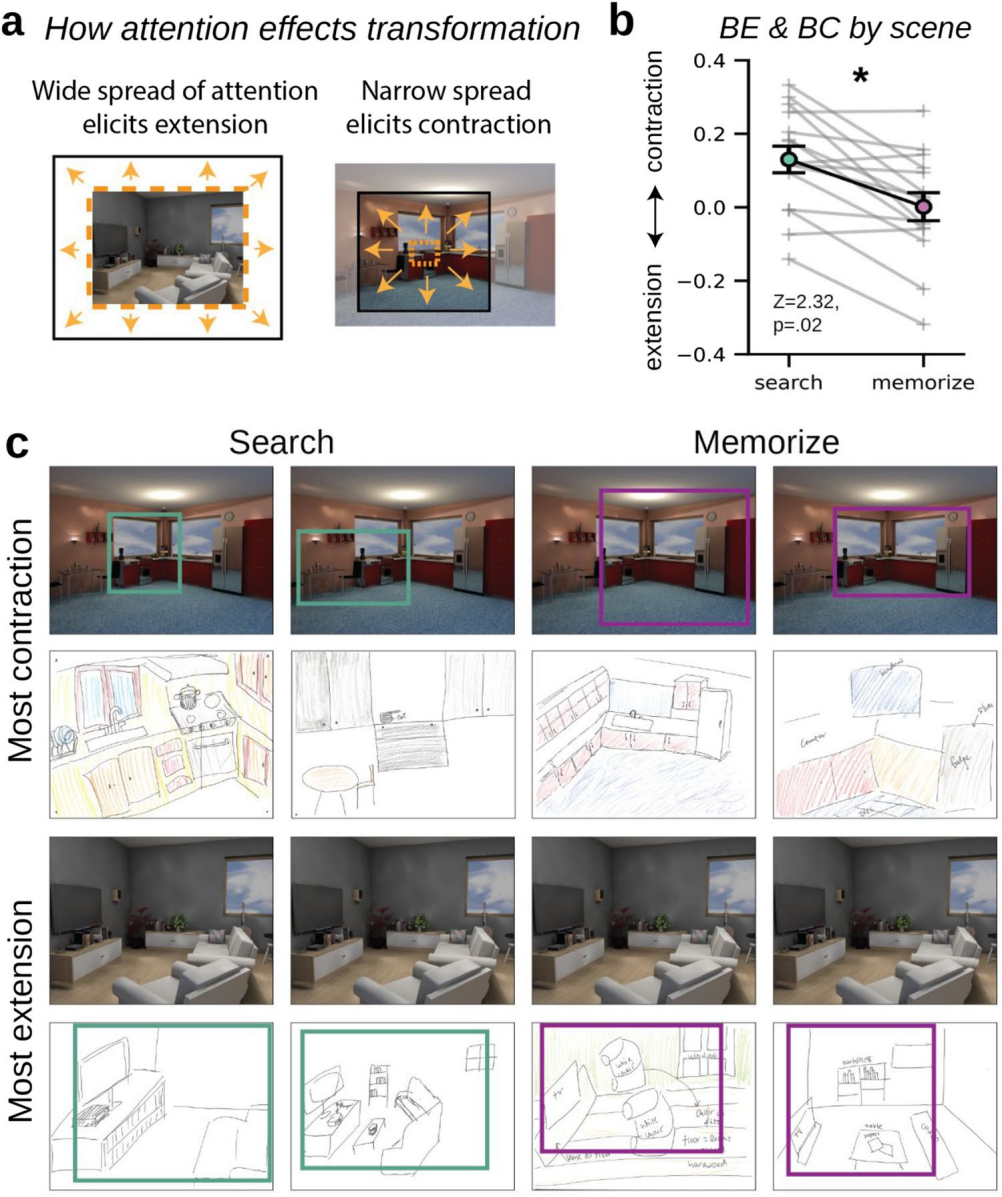
**a** Figure showing how the spread of attention at encoding can influence boundary transformations. The orange box is the spread of attention, and the black box is what the participant remembers of the scene. When the spread of attention is narrow, boundaries could extend beyond the target region in memory, but because the target is a relatively small proportion of the image, a majority of the scene is forgotten. **b** Plot of the average boundary transformation rating across drawings by image. Each gray line represents one of the 15 scene images. Error bars represent the standard error of the mean. Drawings of scenes done in the search condition showed significantly more boundary contraction on average. **c** Example drawings of the scenes that elicited the most boundary contraction and boundary extension for the search (left) and memorize (right) conditions. For drawings with the most contraction (top), the colored outlines on the images show how much of the scene the participant recalled. For drawings with the most extension, the colored outlines on the drawings show the boundaries of the studied image. Area outside of the boundaries is what was extended in memory. (Color figure online)

The main question of this study was whether the patterns of object-based attention used in search would elicit contraction effects in memory above the rate elicited by the image alone. To investigate this, we had an experimentally naïve group of online scorers rate the degree of boundary transformation in the search and memorize drawings. We visualized what percentage of drawings from each condition showed either boundary contraction or extension (Fig. 3). To start, we found that a majority of search drawings had contracted scene boundaries. On average, 62.29% of Search drawings showed boundary contraction, while only 30.73% showed boundary extension. The results from a chi-square test of independence confirmed that the difference between proportions was significant, χ^2^(1,* N* = 283) = 28.0, *p* < .001. Comparatively, only 44.67% of the drawings from the Memorize condition showed boundary contraction, with 47.95% of drawings showing boundary extension, and the chi-square test revealed no significant difference between the proportions, χ^2^(1, *N* = 232) = 0.04, *p* > .5. The transformations ratings for the memorize drawings are consistent with the findings of Bainbridge and Baker (2021), who revealed that scene images have a fairly equal probability of eliciting either contraction or extension. To assess the reliability of the ratings we conducted a split-half analysis across 1,000 iterations and applied the Spearman–Brown correction formula (Fig. 3). Boundary transformation ratings were highly consistent across raters’ responses for both the search (⍴* = 0.64; *p* < .001) and memorize drawings (⍴* = 0.51; *p* < .001). We then looked at the difference in boundary contraction scores averaged by scene image across conditions (Fig. 3b). This analysis allows us to directly compare the effect of the task on memory, as both groups of participants studied the scenes for roughly the same amount of time (Fig. 4). Results from a non-parametric Wilcoxon rank-sums test (WRST) confirmed that scene images were significantly more likely to elicit boundary contraction in memory when participants engaged in target search during the encoding period (*N* = 15, *Z* = 2.32, *p* = .020). Taken together, these findings suggest that object-based attention during scene perception can elicit boundary contraction in memory.

### Smaller spread of attention in Search leads to less memory for other objects

**Fig. 5:**
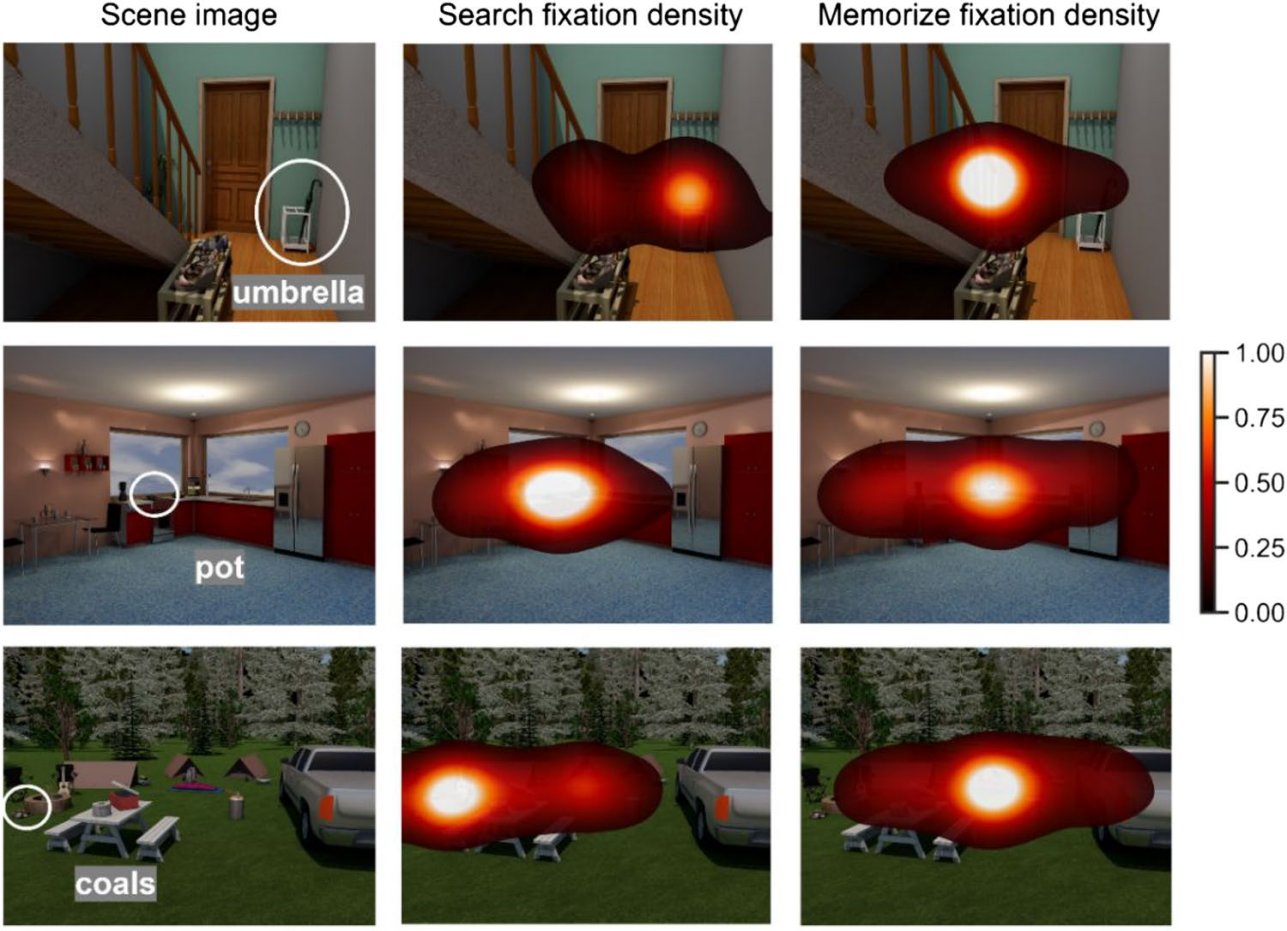
Heatmaps of the average fixation density across participants in the search and memorize conditions. Heatmaps are scaled to a range of 0 to 1. Examples for 3 of the 15 scenes are shown here, with the target object circled in white and labeled by the target cue. Heatmaps show the general tendency for participants in the Search condition to spend more of the trial fixating the target object, while participants in the Memorize condition tended to show a strong center bias in their eye movements. (Color figure online)

Participants exhibited strong task-based influences on their eye movements during study (Fig. 5). Participants in the memorize condition foveated 4.41% of each scene (*SD* = 0.47) on average and exhibited a strong bias to fixate the center of the image. Participants in the search condition foveated significantly less of each scene on average (*N* = 15, *Z* = 3.3, *p* < .001), with their fixations covering only 3.22% of the scene on average (*SD* = 0.89). As expected, participants in the search condition spent significantly more time on the target object (*Z* = 5.78, *p* < .001), fixating it 18.97% (*SD* = 0.09) of trial time on average, while participants in the memorize condition spent only 3.58% (*SD* = 0.02) of the trial time looking at the target (from the search condition).

**Fig. 6:**
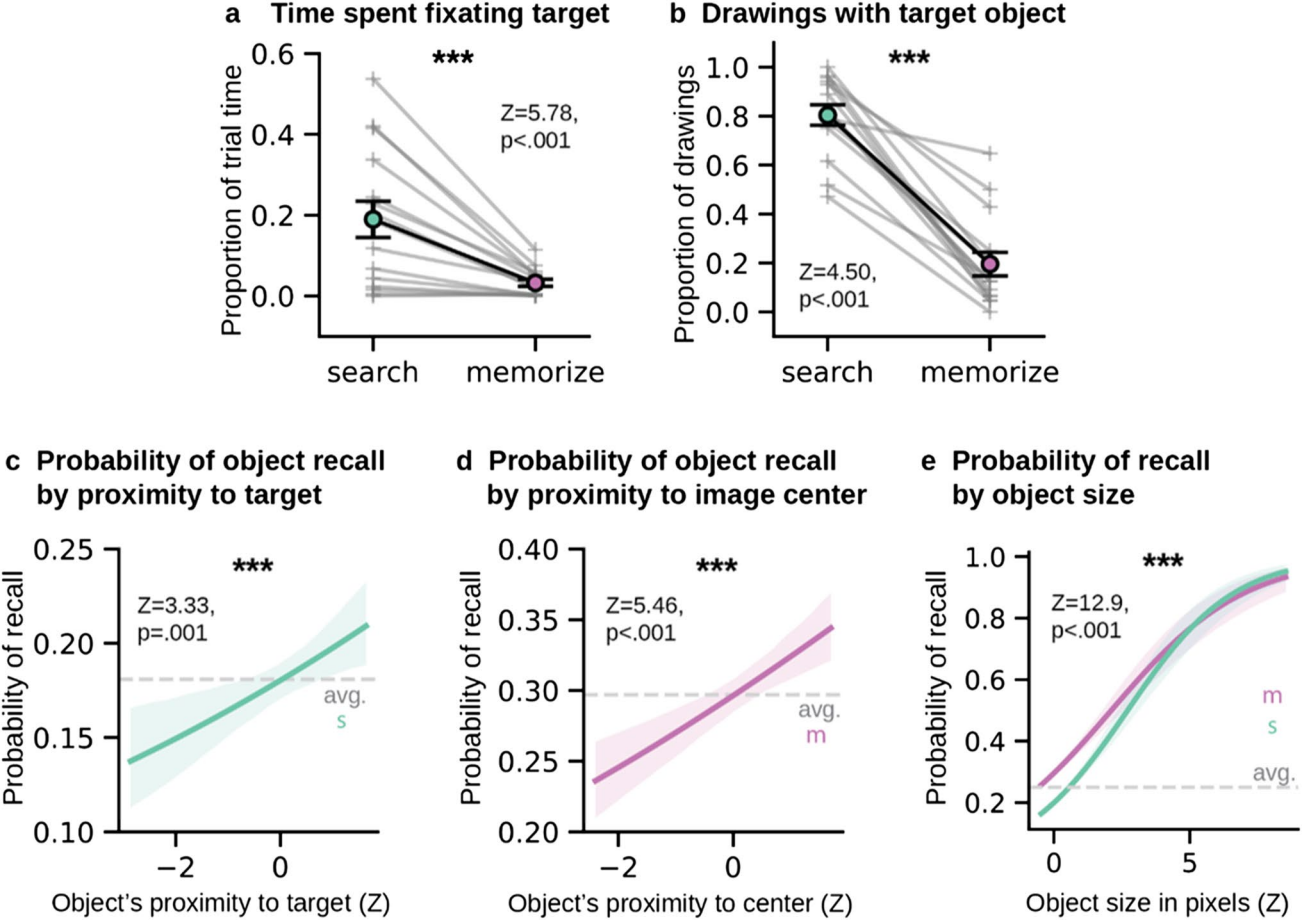
**a.** Plot of the proportion of trial time spent fixating the target object. Participants in the search condition spent a significantly greater proportion of trial time fixating the target. **b.** Plot of the proportion of drawings that included the target object. A significantly greater proportion of search drawings contained the target object. Each gray line represents one of the 15 scene images. Error bars represent the standard error of the mean. **c.** Regression line indicating the probability of recalling an object in the Search condition by its proximity to the target object. **d.** Regression line indicating the probability of recalling an object in the Memorize condition by its proximity to the image center.** e.** Regression lines indicating the probability of recalling an object by its size and condition. Shaded error bars are the confidence interval bootstrapped across 1,000 iterations. (Color figure online)

Given that the target object was more likely to be fixed during encoding in the search condition compared with the memorize condition, we next looked to confirm that target objects would be more likely to be included in drawings from the search group (Fig. 6b). To do so, we showed a separate group of online workers each drawing alongside the original stimulus image and asked them to make a judgment as to whether an outlined object on the image was present in the drawing. From these judgments, we found that the target object was present in 80.36% (*SD* = 16.09%) of search drawings. In comparison, drawings from the memorize group, contained the target only 19.39% (*SD* = 18.85%) of the time (*N* = 15, *Z* = 4.50, *p* < .001), although they included significantly more of the nontarget objects in the scene (memorize: *M* = 29.58%; search: *M* = 18.21%; WRST: *N* = 15, *Z* = 2.76, *p* < .006).

### Distance from the search target predicts memory for nontarget objects

We next tested whether objects included in drawings from each group could be predicted by the location of the target, or the center of the image. Finding that objects in the search condition are more likely to be drawn if they are close to the target would be consistent with a model of memory representations contracting around attended objects (Fig. 6c). We fit a logistic regression model on the recall data from drawings done in the search condition (*N* = 6,165 objects [1,115 objects drawn / 5,050 objects not drawn]). Proximity to the target was defined as the distance from the center of the object segmentation to the center of the target object segmentation, *z*-scored across objects. We found that proximity to the target object significantly predicted whether an object would be included in the memory drawing (β_0_ = −1.51, CI [−1.58, −1.45], *Z* = −45.61, *p* < .001; target proximity: β = 0.11, CI [0.05, 0.18], *Z* = 3.33, *p* = .001). These findings suggest that participants from the Search condition may have maintained a representation of the scene in memory that was focused around the target object.

For the memorize drawings (*N* = 5,782 objects [1,717 drawn / 4,065 not drawn]), we found that proximity to the target was negatively correlated with a likelihood to include the object in the drawing (β_0_ = −0.87, CI [−0.93, −0.82], *Z* = −30.01, *p* < .001; target proximity: β = −0.18, CI [−0.24, 0.12], *Z* = −6.07, *p* < .001); proximity to the center of the image was positively correlated with the object being drawn (center proximity: β = 0.16, CI [0.10, 0.22], *Z* = 5.46, *p* < .001). These results likely reflect the fact that participants in the Memorize condition were more likely to fixate and remember information near the center of the image (Fig. 6d). Target objects were placed around the periphery of the scene images, which could explain the negative relationship between target proximity and likelihood of being drawn.

Additionally, drawings from both conditions showed a tendency to include large, space-defining objects. Results of a logistic regression further confirmed that object memory was significantly predicted by both object size (*n* = 12,230 objects [3,055 drawn / 9,175 not drawn]; β_0_ = −0.87, CI [−0.93, −0.81], *Z* = −29.60, *p* < .001; size: β = 0.41, CI [0.35,0.47], *Z* = 12.90, *p* < .001) and the search condition (β = −0.52, CI [−0.61, −0.44],* Z* = −12.05, *p* < .001). The interaction between size and condition was also significant in the model (β = 0.10, CI [0.01, 0.19], *Z* = 2.28, *p* = .022). Looking at a plot of the model fit (Fig. 6e) we see that memorize drawings show a stronger likelihood of including objects that fall within the first five standard deviations of mean object size. The interaction between object size and condition occurs as object size becomes greater than five standards above the mean, when search drawings start to show a greater likelihood of drawing the object from memory.

### Contraction increases the size that target and non-target objects are drawn

The drawings also provided some insight into how participants in the search represented the targets’ size and location in memory. We asked a group of online workers to provide judgements as to an object's height and width by asking them to draw an ellipse around each object in each drawing. We then defined the height and width of a drawn object as the median radii from a set of three judgements. From these judgments, we found that participants in the search condition consistently drew the target objects from memory as wider (*M* = 4.55% wider, *SD* = 2.18% wider) and taller (*M* = 5.87% taller, *SD* = 1.76% taller) than they originally appeared. A paired-sample signed-rank test of the ellipse size between target objects of the images and target objects in the search drawings confirmed that target objects were drawn significantly larger than portrayed in the stimuli images (*N* = 15, *Z* = 3.30, *p* = .001). Nontarget objects in search drawings were drawn 3.84% wider (*SD* = 1.97%) and 4.94% taller (*SD* = 2.36%), a smaller increase in size than that shown for target objects. However, the differences in drawn width or height between target and nontarget were not significant (width: *Z* = 0.5, *p* > .5; width: *Z* = 1.14, *p* > .5), suggesting that the increase in target object size may be mostly due to the contraction effect.

Memorize drawings that included the target object also showed a significant tendency to overestimate the target’s size (wider: *M* = 2.14%, *SD* = 1.94%; taller:* M* = 2.84%, *SD* = 2.02%). However, results from paired sample signed-rank tests of the average increase in target height and width between conditions confirmed that targets were drawn as significantly taller and wider in memory representations from the Search condition compared with those from the memorize condition (taller: *N* = 14, *Z* = 2.44, *p* = .015; wider: *N* = 14, *Z* = 3.63, *p* < .001 [we excluded one scene from this analysis as no drawings from the memorize condition of this scene included the target object]). This result points to the idea that when memory representations for a scene are contracted, objects tend to be remembered as larger (or closer) than they originally appeared (Kirsch et al., 2018). The finding that target objects were drawn as even larger in search drawings compared with memorize drawings lends support to the idea that these drawings represent memories where the boundaries were significantly contracted around the target object.

**Fig. 7:**
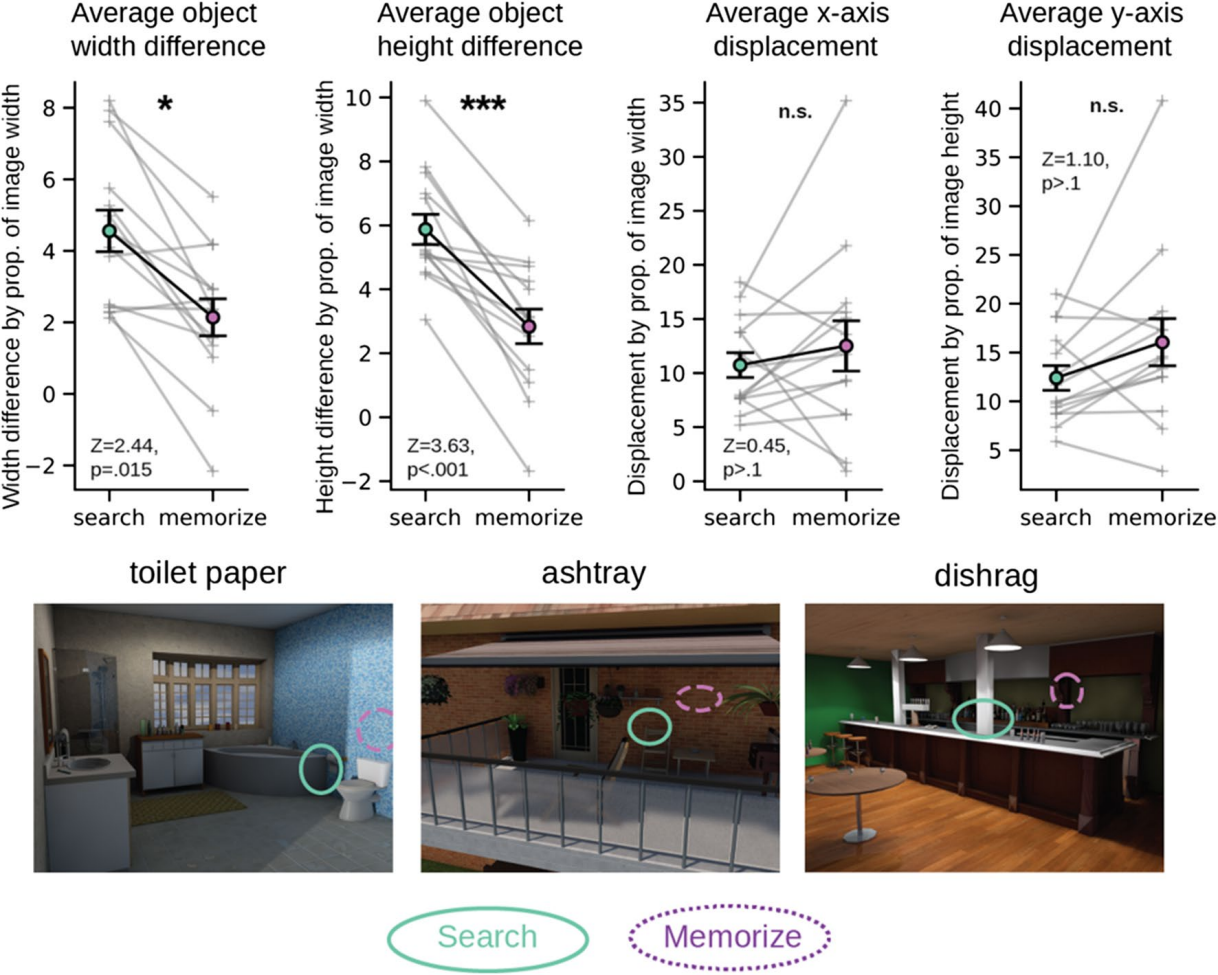
(Top left) The mean height and width difference between objects of the different conditions and objects in the original image. (Top right) The mean *x*-axis and *y*-axis distance between object centroids of the different conditions and object centroids in the original image. Each gray line represents one of the 15 scene images. Error bars represent the standard error of the mean. (Bottom) Example maps of the average ellipse encompassing the target objects by condition. Target objects are noted above the scenes. (Color figure online)

The ellipse judgements provided by the online raters also provided a metric for measuring whether participants had accurate memory for the objects’ locations throughout the scene (Fig. 7). For this analysis we defined object location as the median centroid from a set of three ellipse judgments. Participants in the search condition drew the target objects close to where they appeared in the stimulus image, with target objects centroids displaced on average by 10.74% (*SD* = 4.28%) of the scene overall in the *x*-direction, and by 12.40% (*SD* = 4.73%) in the *y*-direction. A paired-sample signed-rank test found that there was no significant difference in where the object centroid was located on the *x*-axis compared with where it was drawn (*N* = 15, *Z* = 0.97, *p* > .1). A paired-sample signed-rank test of the drawn and real target centroid location on the *y*-axis revealed that there was some significant displacement along that axis (*N* = 15, *Z* = 2.22, *p* = .03). Target objects were displaced slightly more in the memorize drawings (*x*-axis: *M* = 12.51%, *SD =* 8.71%, *y*-axis: *M* = 16.05%, *SD* = 9.03%), although results from paired-sample signed-rank tests found that this difference in *x-* and *y*-axes displacement between conditions was not significant (*x*-axis: *N* = 14, *Z* = 0.46, *p* >.1; *y*-axis: *N* = 14, *z* = 1.10, *p* > .1). The significant degree of displacement along the y-axis for target object in the Search condition and the lack of significant displacement along the *x*-axis is likely due to there being more pixels along the *x*-axis in the stimulus images (the stimuli 1,280 pixels wide × 960 pixels tall). Therefore, we should not rule out that participants in the search condition had some displacement overall for where the target object was located. However, the changes in location could be seen as slight compared with the magnitude of increase in size in memory for target objects.

## Discussion

This study sought to provide insight into how scene memory is shaped by selective attention at encoding. Specifically, we looked to see if instructing viewers to constrain spatial attention to target objects through visual search would impact the magnitude and trend of transformation effects. We hypothesized that the tightly constrained focus of attention needed to efficiently select and recognize targets would lead to significant boundary contraction in memory. Consistent with our hypothesis, we found a high rate of boundary contraction in drawings from participants who engaged in search, above the rate reported in previous studies (Bainbridge & Baker, 2020; M. Greene & Trivedi, 2022; Hafri et al., 2022; Lin et al., 2022; Park et al., 2021). Further, we found that a group of participants who viewed the same scenes for roughly the same amount of time but only to memorize the scenes, had significantly less boundary contraction in their drawings, and in fact showed roughly equal rates of contraction and extension. Taken together these results suggest that focused attention on target objects during encoding can lead to those objects having an exaggerated role in memory, with boundaries contracting around them.

Many studies on boundary transformations manipulate the properties of the image stimuli, but rarely give explicit instructions on where to look. In this study, we instructed participants to look for specific targets in the search condition and found an increased rate of contraction. Target objects were included in four-fifths of all search drawings and the likelihood of any other object being included was commensurate with their distance from the target. This suggests that participants in the search condition centered their memories for the scene around the target object leading to scene contraction around the target object. However, without specific features to guide looking behaviors, as in our memorize condition, viewers tend to show a center bias in their fixations on an image (Bindemann, 2010; Tatler, 2007). Although there was overall less contraction in the memorize condition, contracted drawings showed diminishing memory for objects with increasing distance from the center of the image. This suggests that memory for the scene was constructed around information in the center of the image, although looking was likely highly idiosyncratic. Nevertheless, this overall explanation for our data are consistent with the earliest findings on boundary contraction which showed a loss of peripheral information near the image boundaries (Intraub et al., 1992).

Our work is not the first to reveal a significant relationship between attentional spread and boundary transformations in memory (Intraub et al., 2008). For example, manipulating participants to attend to image edges lessens boundary extension (Gagnier et al., 2013; McDunn et al., 2016). Similarly, perceptual object grouping due to semantic relatedness spreads attention and leads to increased rates of extension in memory (M. Greene & Trivedi, 2022). Inversely, scenes with especially valent objects that capture and hold attention, elicit more boundary contraction (S. A. Christianson, 1984; S.-Å. Christianson & Loftus, 1987; D. M. Green et al., 2019; Ménétrier et al., 2018; Safer et al., 1998, 2002; Takarangi et al., 2016; Wonning, 1994). These findings show that constraining attention to specific objects at encoding leads to increased rates of contraction, whereas distributing attention tends to lead towards greater extension. Our findings are consistent with these studies and provide further evidence that attention plays a pivotal role in eliciting boundary transformations, even when attention is guided by task demands rather than image properties.

Our results also complement work showing that viewers have different rates of transformation in memory as a consequence of whether they attend to scene or object information. For example, boundary extension does not occur without tangible (McDunn et al., 2014) or implied (Intraub et al., 1998) scene background information, and boundary contraction is more likely to occur for images of objects on blank backgrounds (Gottesman & Intraub, 2002; Intraub et al., 1998). Other work on memory for images of individual objects show a pattern of conversion where remembered objects are transformed towards their real-world size (Konkle & Oliva, 2007; Lin et al., 2022), possibly reflecting a closer preferred viewing distance for processing object information (Bainbridge & Baker, 2020; Chen et al., 2022; Park et al., 2021). Visual processing of objects and scenes operates through different functional pathways (Doshi & Konkle, 2023; Park et al., 2023) and this appears to determine the directionality of boundary transformation.

More work is needed to determine if attention contributes to other sources of memory transformation or not. For example, natural statistics of scene depth are strongly correlated with the trend and magnitude of transformation (Lin et al., 2022), with images with unnaturally deep depth of field having a higher likelihood of eliciting boundary contraction (Gandolfo et al., 2023). Likewise, judgements of perspective distance have been shown to be highly reliant on patterns of spatial frequency (Barron et al., 2022; Brady & Oliva, 2012; Lescroart et al., 2015; Oliva & Torralba, 2001; Oliva et al., 2006). These results may be related to attentional effects, such as a strong central fixation bias, or they may be due solely to perceptual processes. A better understanding of the multiple possible mechanisms underlying boundary transformations in memory will require further work on how the brain processes peripheral versus foveal, eccentric, object and scene information, such as that being done by Konkle and colleagues (Doshi & Konkle, 2023; Julian et al., 2016; Park et al., 2023). Discovering how the neurobiological properties of the visual system contribute to memory transforms will likely relate to the multisource account of boundary extension as a “filling-in” of expected information (Gottesman & Intraub, 2002; Intraub, 1997, 2002, 2010; Intraub et al., 1992; Intraub & Berkowits, 1996; Maguire et al., 2016; Mullally et al., 2012; Park et al., 2007).

In summary, we believe that our results set-up an interesting account for how selective attention in visual processing can drive transformation effects. We found that requiring the participants to focus attention in order to identify objects during encoding led memory drawings to show a higher rate of boundary contraction than found in those of participants not required to constrain attention. We propose that attentional guidance in part determines the trend and magnitude of boundary transformation effects in memory, along with other competing factors, such as the distance and image contents. Together these findings contribute to the evolving discovery of what factors influence transformation effects in memory.

## Data Availability

Drawings and code will be made publicly available on OSF link.

## Declarations

### Open practices statement

Drawings and code will be made publicly available on the Open Science Framework (https://osf.io/mkas7/). The design for this study was preregistered on As Predicted (https://aspredicted.org/zf397.pdf). Analyses on the how search leads to memory for size and location of the target object, and memory for other objects in relation to the target object, were preregistered.
