# Correction: Object-based attention during scene perception elicits boundary contraction in memory

**DOI:** 10.3758/s13421-024-01574-z

**Published:** 2024-05-10

**Authors:** Elizabeth H. Hall, Joy J. Geng

**Affiliations:** 1 Department of Psychology, University of California Davis, Davis, CA 95616 USA; 2 Center for Mind and Brain, University of California Davis, Davis, CA 95618 USA

**Correction: Memory & Cognition**

10.3758/s13421-024-01540-9

In the section of the article under the heading Contraction increases the size that target and non-target objects are drawn, please note the following correction of a sentence of text from the article as originally published.

“The results of our recent study confirmed this prediction for size perception: when attention was allocated at the center of a circular stimulus, that stimulus was perceived as larger as compared with a neutral attentional condition (Kirsch et al., 2018).”

should read:

“This result points to the idea that when memory representations for a scene are contracted, objects tend to be remembered as larger (or closer) than they originally appeared (Kirsch et al., 2018).”

In the first paragraph of the same section, the following text correction should be noted:

“Nontarget objects in search drawings were drawn 4.55% wider (SD = 2.18%) and 5.87% taller (SD = 1.76%), a smaller increase in size than that shown for target objects.”

should read

“Nontarget objects in search drawings were drawn 3.84% wider (SD = 1.97%) and 4.94% taller (SD = 2.36%), a smaller increase in size than that shown for target objects.”

The original article has been corrected.

